# Discutient Application to the Indurated Epididymis

**Published:** 1859-04

**Authors:** 


*Discutient Application to the Indurated Epididymis.*—Extract ot belladonna, one drachm and a half; soften in from fifteen to twenty drops of water, and add one drachm and a half of tincture of iodine. The effect is both sedative and discutient.—Dr. Diday.

